# GRANDPARENTING CHILDREN WITH DISABILITIES AND ITS IMPACT ON GRANDPARENT HEALTH

**DOI:** 10.1093/geroni/igz038.2341

**Published:** 2019-11-08

**Authors:** Ynesse Abdul-Malak, Madonna Harrington Meyer

**Affiliations:** 1 Colgate University, Hamilton, New York, United States; 2 Syracuse University, Syracuse, New York, United States

## Abstract

Across the US, millions of grandparents are providing vital care for their grandchildren with disabilities when their adult children are in need of assistance and public programs do not provide needed supports. Research suggests the impact on grandparent physical health is mixed. This paper draws on in-depth interviews with 50 grandparents to explore how caring for grandchildren with disabilities shapes their physical wellbeing. We use life course perspective to assess the choices grandparents make from available resources and options at different stages of their lives and the effects on their health. We find in addition to providing routine care, helping with feeding, bathing, and dressing, some grandparents provide constant supervision and medically intense care, such as tending to feeding tubes, catheters, and oxygen lines. Many grandparents said care work has improved, or at least helped sustain, their overall level of fitness, while other grandparents find that care work has adverse impacts. Often there is too much chasing, bending, and lifting for their aging bodies. This paper suggests that stronger social programs for children with disabilities and the grandparents who step in to help them would decrease the negative effects of care work on grandparent health.

